# Supplementary material for: Learning curve and surgical outcome of robotic assisted colorectal surgery with ERAS program
Source: Sci Rep. 2022 Nov 29;12:20566. doi: 10.1038/s41598-022-24665-w (PMC9709162; doi:10.1038/s41598-022-24665-w)
Supplement: Supplementary file 1 — Supplementary Information 1. [file 41598_2022_24665_MOESM1_ESM.zip › LSC Raw data-Ñ[▒K/078/N_ñw╝╨░O▒Kñσ.pdf]

W52 / W52

臺 中 榮 民 總 醫 院
手術室護理記錄

[redacted]

基本資料

- 室別： 開刀房-18室 ，手術科別： CRS ，手術類別： 常規 ，日期： 2020/04/24
- 進入時間： 0745 ； 麻醉開始時間： 0745 ； Time Out時間： 0850 ； 劃刀時間： 0851 ； 手術結束時間： 1210 送出時間： 1225 ；
- 麻醉方式： GE

  診斷：NPH s/p L-P shunt

  手術名稱：1.Laparoscopic Anterior resection and anastomosis - malignant

  2.Removalof subscutaneous, in muscle or deep foreign body

  手術局部用藥：NIL

  術前預防性抗生素：
  - 抗生素名稱一： Veterin 1GM IVD/由麻醉護理師執行
  - 時間： 0840
  - 上次簽章: 呂佳旻

  ，追加預防性抗生素：
- 手術醫師： 黃愉芬 ，蔣鋒帆 ，林昌霖 ，楊曙榮
- 麻醉醫師： VS 李富榮
-
  - 刷手護士A ： 黃珮涵 ，時間： 0745 ，接替刷手一： 陳家瑋 ，時間： 1200
  - 流動護士A ： 呂佳旻 ，時間： 0745 ，接替流動一： 陳妍希 ，時間： 1100

皮膚狀況

A-V Shunt： 無

術前： 完整

記錄者： 上次簽章: 呂佳旻

術後： 完整

記錄者： 上次簽章: 陳妍希

手術準備

手術臥式 單一手術臥式

-
  - 手術臥式： 截刀臥式
  - 使用附件： 溫毯 38 ℃ ，矽膠枕頭 ，其它： 腳架
  - 皮膚準備： 其它： 2% Chlorhexidine
  - 電燒： 使用電燒: 電極片位置： 右大腿 ，記錄者： 上次簽章: 呂佳旻

止血帶T： 無

植入物

無

標本送檢

- 細菌培養 無

- 細胞學 無
- 冰凍切片 無
- 病理標本
  組織名稱一： IMA root
  ,
  組織名稱二： distal cut end
  ,
  組織名稱三： rectum
- 截肢 無

標本交付 標本： 3 袋 ，標本： 0 盒 ，標本： 0 瓶 ，細胞學： 0 管 ，Culture Tube： 0 管 ，被交付醫師： CR 楊曙榮 ，

- 記錄者： 上次簽章: 陳妍希
- 時間： 1210

傷口術後敷料

傷口敷料： 傷口敷料： 無

- 請於圖上標示部位：

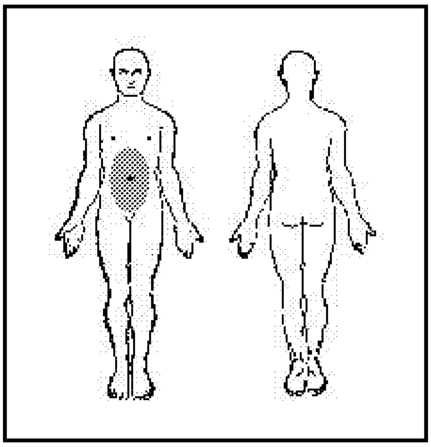

- 傷口敷料： Gauze

導管

導管	天數	導管資訊	插入日期	插入時間	更換日期	拔除日期	拔除時間	拔除原因	預計拔除/更換	預計拔除/更換日期
Sump NG Tube (1)	1	號碼:16 Fr.; 深度:50cm; 部位:胃;	2020/04/24	0840						
Lumboperitoneal Shunt(1)	1	部位:脊椎;	2020/04/24	1150						
A-line(1)	1	號碼:20 G; 部位:右手;	2020/04/24	0920						
Penrose Drain (1)	1	號碼:12 mm.; 部位:骨盆腔;	2020/04/24	1130						
Foley(1)	1	號碼:16 Fr.; 部位:膀胱;	2020/04/24	0830						

用物計數

- 手術中出血量： minimal c.c.
- 手術中尿量： 200 c.c.
-

- ○ 其它： 無

用物計數： 多切口同時縫合

- 1.器械： 正確 ，2.縫針： 正確 ，3.刀片： 正確 ，4.紗布： 正確 ，5.腹部墊： 未使用 ，6.紗球： 未使用 ，7.PEANUTS ： 未使用 ，8.紗條： 未使用 ，9.絲帶： 未使用 ，10.針頭： 未使用 ，11.Vessel loop： 未使用 ，12.Boots： 未使用 ，13.Jelco： 未使用 ，14.棉條： 未使用 ，
  - 第一次：
    - 刷手護士 ： 黃珮涵
    - 流動護士： 上次簽章: 陳妍希
    - 時間： 1140

  ,
  - 第二次：
    - 刷手護士 ： 黃珮涵
    - 流動護士： 上次簽章: 陳妍希
    - 時間： 1145

  ,
  - 第三次：
    - 刷手護士 ： 黃珮涵
    - 流動護士： 上次簽章: 陳妍希
    - 時間： 1150

備註

備註： NIL

- 記錄者： 上次簽章: 呂佳旻
- 時間： 1100

填寫護理人員 陳妍希

正式摘要

W52 / W52

doctorRank:B
